# Unexpressed but Indispensable—The DNA Sequences That Control Development

**DOI:** 10.1371/journal.pbio.0030019

**Published:** 2004-12-02

Amidst the hoopla over the exact number of genes we have in our genome—more than a fruitfly, fewer than a rice plant—a more fundamental genetic truth has often been obscured. The expression of 20,000–30,000 genes is under the control of an uncounted host of non-coding sequences, which bind transcription factors and thereby regulate when and where genes are expressed. Unlike coding sequences, whose signatures are easy to spot, the characteristic features of non-coding regulatory elements are largely unknown, making their discovery by simple sequence analysis difficult. In this issue, Greg Elgar and colleagues attack this problem by comparing the non-coding sequences of the human and the pufferfish.

Since the last common ancestor of these two species existed 450 million years ago, the authors reasoned that non-coding sequences conserved between them are likely to be fundamental to vertebrate development. Through sequence alignment with increasingly strict criteria, they identified 1,373 highly conserved non-coding elements (CNEs), with an average length of about 200 base pairs. The average sequence match is 84%: not perfect, but much higher than for coding regions shared by humans and pufferfish. A quick check showed that virtually all the sequences also occurred in rodents, chickens, and zebrafish, but not in the nematode, fruitfly, or even the sea squirt, a primitive non-vertebrate chordate.[Fig pbio-0030019-g001]

**Figure pbio-0030019-g001:**
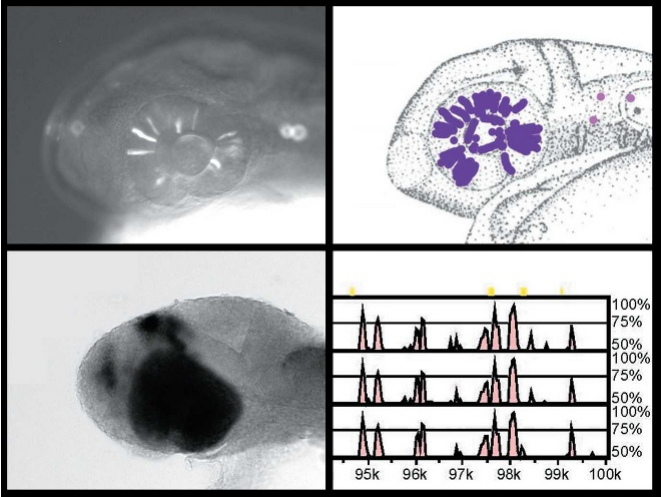
Highly conserved vertebrate non-coding elements direct tissue-specific reporter gene expression

CNEs are not spread uniformly throughout the genome. Instead, they are bunched together in fewer than 200 clusters, most of them in close proximity to genes implicated in transcriptional regulation or development. This clustering of CNEs suggests they may not only attract transcription factors, but may also influence the local topology of the DNA, thereby controlling access to their associated gene. Several clusters also appear in regions without any known genes—the identification of these clusters might lead to the discovery of new developmentally significant genes.

While “in silico” discoveries such as this can be the jumping-off point for whole new areas of investigation, their validity must be tested “in aqua,” in the wet biology of real organisms. For this Elgar and colleagues chose the zebrafish, because its transparent embryo is ideal for observing developmental events. They injected individual CNEs into embryos, along with a green fluorescent protein (GFP) reporter. By day two of development, 23 out of 25 CNEs injected had upregulated GFP expression, indicating interaction of these sequences with endogenous transcription factors. Different CNEs caused different regional patterns of expression, in keeping with their presumed roles in distinct developmental processes.

The discovery of these developmentally important sequences opens several avenues of new research. For example, analyzing the sequence and location of these CNEs may help point the way to other non-coding elements that remain undiscovered. It is also likely that mutations in these critical sequences cause human diseases. Studying how such mutations drive development astray may lead to better understanding not only of these diseases, which are likely to be rare, but also of normal human development.

